# The expression of miRNA-216b is negatively correlated with 18F-FDG uptake in non-small cell lung cancer

**DOI:** 10.1186/s12957-021-02376-2

**Published:** 2021-09-01

**Authors:** Mingfei Zuo, Lan Yao, Lijuan Wen, Jianfei Shen, Na Zhang, Tian Bai, Qicheng Huang

**Affiliations:** 1 Imaging Center, The Third Affiliated Hospital of Qiqihar Medical University, No. 27 Taishun Street, Tiefeng District, Qiqihar, 161002 Heilongjiang China; 2 Department of Nuclear Medicine, The Third Affiliated Hospital of Qiqihar Medical University, Qiqihar, 161002 Heilongjiang China

**Keywords:** miRNA-216, Non-small cell lung cancer, PET/CT, Adenocarcinoma, Squamous cell carcinoma

## Abstract

**Background:**

This study aimed to investigate the correlation between miRNA-216b expression in patients with non-small cell lung cancer (NSCLC) and ^18^F-fluorodeoxyglucose (FDG) uptake by PET/CT and to explore the clinical application value of 18F-FDG PET/CT in miRNA-216b based on therapy for NSCLC.

**Methods:**

Eighty patients with NSCLC and 40 healthy subjects were enrolled in our study. The SUVmax of the lesion area by PET/CT imaging was calculated. SUVmax represented the highest concentration of 18F-FDG in the lesion. The expression of miRNA-216b in the plasma and fiber bronchoscopic puncture of NSCLC patients was detected by RT qPCR. Then Pearson correlation analysis was used to analyze the correlation between miRNA-216b expression and 18F-FDG uptake in patients with different types of NSCLC.

**Results:**

Compared with healthy subjects, SUVmax of early adenocarcinoma and advanced adenocarcinoma were increased. Compared with healthy subjects, SUVmax of early squamous and advanced squamous were increased. And the SUVmax content of advanced adenocarcinoma and squamous cell carcinoma was higher than that of early adenocarcinoma and squamous cell carcinoma. Compared with healthy subjects, the expression of miRNA-216b in the plasma of patients with early and advanced adenocarcinoma was reduced, and the expression of miRNA-216b in the plasma of patients with early and advanced squamous cell carcinoma was reduced. Compared with adjacent tissues, the expression of miRNA-216b in early adenocarcinoma tissues and advanced adenocarcinoma tissues was reduced, and the expression in early squamous cell carcinoma and advanced squamous cell carcinoma was reduced. Pearson correlation analysis showed a negative correlation between SUVmax and miRNA-216b (plasma and tissue) in patients with four types of NSCLC.

**Conclusion:**

miRNA-216b expression was negatively correlated with ^18^F-FDG uptake in NSCLC. miRNA-216b could be used for the classification and staging of non-small cell lung cancer. ^18^F-FDG PET/CT may be used to evaluate the therapeutic response in application of miRNA-216b-based cancer treatment.

**Supplementary Information:**

The online version contains supplementary material available at 10.1186/s12957-021-02376-2.

## Background

Lung cancer is a serious global health problem. Statistics on cancer-related deaths show that lung cancer accounts for 25% of male and 15% of female deaths. It is the first leading cause of cancer death in men and the second leading cause in women [1–5]. According to histological classification, lung cancer is divided into two types: non-small cell lung cancer (NSCLC) and small cell lung cancer [6]. NSCLC accounts for 75–85% of lung cancer [7]. Therefore, it is important to explore the molecular mechanism of the occurrence and development of NSCLC, to determine its correlation with imaging, in order to improve its detection rate and diagnostic accuracy. Positron emission tomography (PET) can detect the distribution of positron-emitting radionuclides in the body [8]. Short-metabolizing radioisotopes (radionuclides) are used in tumor PET imaging. The most commonly used radionuclide, ^18^F, can label glucose to produce ^18^F-fluorodeoxyglucose (^18^F-FDG). ^18^F-FDG is a glucose analog that can be transported into cells by glucose transporters on the cell membrane and catalyzed by hexokinase to become glucose 6-phosphate. However, glucose 6-phosphate cannot enter the tricarboxylic acid cycle to participate in biochemical metabolism. It retains in cells, then deposits in tissues. Glycolysis process of most malignant tumors are more active than normal tissues (called the Warburg effect), so the number of glucose transporters on tumor cell membranes is larger, and the activity of hexokinase inside cells is increased, and the ability to take up and concentrate ^18^F-FDG is significantly enhanced. Therefore, we take advantage of this feature of malignant tumor cells to label them with ^18^F-FDG. And then, PET imaging is used to diagnose the tumor.

In recent years, miRNAs have been reported to participate in the development of cancers [9–11]. A large amount of literature shows that tumor-specific miRNAs and their direct target genes play an important role in the carcinogenesis and progression of non-small cell lung cancer, which may provide diagnostic and therapeutic targets for patients [12–15]. Among them, miRNA-216b has been found to be involved in the development of various cancers, and a large number of reports have confirmed that the expression of miRNA-216b is significantly reduced in cancer tissues and plasma. These findings indicate that miRNA-216b is expected to be a tumor diagnostic marker [16]. miRNA-216b is reported to regulate the proliferation and invasion of NSCLC by targeting SOX9 and is an important tumor suppressor in NSCLC [17]. There are literature data using 18F-FDG microPET-CT scanning to evaluate the efficacy of miR-143 on poorly differentiated thyroid cancer. The results showed that the uptake of 18F-FDG in tumors was reduced, which corresponded to the downregulation of Hexokinase 2 (HK2) expression in tissues. Results suggested that miR-143 could be used to specifically assess the therapeutic efficacy of advanced thyroid cancer xenografts by 18F-FDG-microPET/CT [18]. Therefore, we considered whether the expression of miRNA-216b in NSCLC patients was related to the uptake of 18F-FDG. At present, relationship between miRNA-216b and the uptake of 18F-FDG is not clear. Therefore, in this work, we aimed at exploring the correlation between miRNA-216b expression and 18F-FDG uptake in early and advanced NSCLC (squamous cell carcinoma and lung adenocarcinoma) patients. We also tried to clarify the role of FDG PET scans in assessing treatment response in a next era where miR-216b addressed therapy could be available.

## Methods

### Patients

We retrospectively analyzed 80 patients with NSCLC who underwent radical surgery for lung cancer in our hospital between January 2016 and January 2019. There were 50 men and 30 women, aged 35–65 years, with a median of 40.6 years. Inclusion criteria for the patients were pathological diagnosis confirmed as lung adenocarcinoma, squamous cell carcinoma, or adenosquamous carcinoma; completed PET/CT images, tissue specimens, and clinicopathological data; clinical confirmation of no other malignant lesions; and no radiotherapy or chemotherapy before surgery. The study comprised of 40 cases of adenocarcinoma (20 in early stage and 20 in advanced stage) and 40 cases of squamous cell carcinoma (20 in early stage and 20 in advanced stage). All patients were divided into four groups according to the criteria of the 7th edition of the American Joint Committee on Cancer: early squamous cell carcinoma group, advanced squamous cell carcinoma group, early adenocarcinoma group, and advanced adenocarcinoma group.

The control group included 40 healthy subjects: 25 men and 15 women who were randomly selected from our hospital, aged 35–65 years, with a median age of 50 years. A series of tests was performed on healthy subjects, including routine blood, urine, and stool examination; liver and kidney function tests; chest radiography; tumor marker test; and CT and magnetic resonance imaging. Subjects with no significant abnormalities, no high blood pressure, diabetes, and other chronic medical history were included. Subjects with any of the above abnormalities were excluded. The study was approved by the Ethics Committee of our hospital and all patients signed informed consent forms.

### PET/CT imaging method

Patients underwent PET/CT before treatment. They were fasted for at least 6 h before the test, and fasting blood glucose was measured and controlled below 6 mmol/L. Patients were intravenously injected with ^18^F-FDG at 5.2 MBq/kg body weight, and rested for 1 h, and then systemic imaging was performed with a maximum intensity of 370 MBq (10 mCi). Whole PET body tomography (two-dimensional scanning, average 6 or 7 beds, 4 min/bed) was performed using the uMI 510 PET/CT instrument (Shanghai United Imaging Medical Technology Co. Ltd, China), and the radiochemical purity of ^18^F-FDG was > 95%. The intensity of ^18^F-FDG uptake was expressed as a standard uptake value (SUV). SUV = radioactive concentration of the lesion (kBq/ml) / injected dose (MBq) / body weight (kg). The maximum SUV (SUV_max_) represented the highest concentration of ^18^F-FDG in the lesion. Enhanced CT was performed prior to PET (scan parameters: 140 kV, 90 mA, pitch 0.75, tube speed 7.5 s/rot, layer thickness 5 mm). To minimize radiation, CT was adjusted to 76–151 mAs depending on the patient’s weight. In order to match the PET scan, spiral CT was performed by filtering back projection to obtain 512 × 512 pixel images with a slice thickness of 5 mm.

### PET/CT image analysis

All PET/CT examinations were read by at least three radiologists with several years of work experience and relevant PET/CT training. The radiologists identified tumor lesions by visual and SUV_max_ methods in a blinded manner and independently assessed the scans based on the location, size, morphology, degree of concentration, uniformity of radioactivity distribution, and relationship with adjacent tissues. Based on the location of the lesion shown on CT, the regions of interest without corresponding radioactivity concentration abnormalities in PET images and with the same lesion size was selected, and the SUV_max_ of the lesion region was calculated by a specific program.

### Detection of miRNA-216b expression in plasma

When detecting the expression of miRNA-216b in the plasma of NSCLC patients, healthy subjects were used as controls. mirVana PARIS kit (Beijing Tiangen Biochemical Technology Co., Ltd.) was used to obtain plasma miRNAs from patients and healthy subjects. Total RNA was reverse transcribed into cDNA. The reverse transcription reaction system consisted of 4 μL 5× reverse transcription buffer, 0.75 μL dNTP (10 mM), 1.2 μL primer, 0.2 μL reverse transcriptase, 3 μg RNA, and double-distilled water. The cycling conditions for reverse transcription were as follows: 25 °C for 30 min, 42 °C for 30 min, and 85 °C for 5 min. For measurement of miRNA-216b expression level, 20-μL reaction system for quantitative PCR consisted of 10 μL SYBR-Green I mixture, 2 μL forward primer, 2 μL reverse primer, 2 μL cDNA, and 4 μL double-distilled water. The thermal cycling conditions for real-time quantitative PCR were as follows: 95 °C for 30 s, 40 cycles of 95 °C for 5 s, and 60 °C for 30 s. The primer sequences used are shown in Table 1. The RT qPCR results were calculated using the 2^−ΔΔCT^ method. Three copies of each sample were analyzed.

**Table 1:** Real-time quantitative PCR experimental primer sequences

Gene	Sequence (5′ to 3′)
miRNA-216b	F: GCC GCG CTA AAG TGCTTA TAG TG
	R: CAC CAG GGT CCG AGGT
U6	F: TGC GGG TGC TCG CTT CGG CAGC
	R: CCA GTG CAG GGT CCG AGGT
miRNA-145	F: CAG TCT TGT CCA GTT TTC CCAG
	R: TAT CCT TCT TCT CCT CTC TCT CTC
miRNA-28-3p	F: CGG ATC CAG GCC CTT CAA GGA CTT TCT
	R: CGA ATT CAC AGA GCT CCT GCT GTG TCA

U6, miRNA-145, and miRNA-28-3p were selected as the internal reference gene when detecting the expression of miRNA-216b. Real-time quantitative PCR results were calculated using the 2^−ΔΔCT^ method, and each sample was repeated three times

### Lung cancer tissue collection and miRNA 216b expression

When detecting miRNA-216b at the center of neoplasms in NSCLC, adjacent tissues 1 cm from tumor margin were selected as controls. Fiber bronchoscopic puncture was performed. At the puncture site, 20 mL was sucked by the puncture needle using a syringe for 20 s, and the procedure was performed three times for each subject. Total RNA was extracted from tissue samples using TRIzol reagent (Shanghai Huiying Biological Technology Co. Ltd.). A total of 2 μL RNase-free DNase I (Beijing Baiao Laibo Technology Co. Ltd.) was used to remove the DNA. Total RNA was reverse transcribed into cDNA. The reverse transcription reaction system consisted of 4 μL 5× reverse transcription buffer, 0.75 μL dNTP (10 mM), 1.2 μL primer, 0.2 μL reverse transcriptase, 3 μg RNA, and double-distilled water. The cycling conditions for reverse transcription were as follows: 25 °C for 30 min, 42 °C for 30 min, and 85 °C for 5 min. For measurement of miRNA-216b expression level, 20-μL reaction system for quantitative PCR consisted of 10 μL SYBR-Green I mixture, 2 μL forward primer, 2 μL reverse primer, 2 μL cDNA, and 4 μL double-distilled water. The thermal cycling conditions for real-time quantitative PCR were as follows: 95 °C for 30 s, 40 cycles of 95 °C for 5 s, and 60 °C for 30 s. The primer sequences used were shown in Table 1.

### Statistical analysis

The experimental data were processed and analyzed by GraphPad Prism and SPSS 24.0 software, and the data were expressed as mean ± standard deviation. When the data had a normal distribution, *t* tests were used for comparison between two groups. Correlation analysis of PET/CT SUV_max_ and miRNA-216b expression levels was performed using Pearson correlation. *P* < 0.05 indicated that the difference was statistically significant, and *P* < 0.01 indicated that the difference was highly significant. The larger the absolute value of the correlation coefficient, the stronger the correlation: the closer the correlation coefficient was to 1 or 1, the stronger the correlation. The closer the correlation coefficient was to 0, the weaker the correlation. Normally, the correlation strength of the variables is judged by the following range of values: correlation coefficient 0.8–1.0, extremely strong correlation; 0.6–0.8, strong correlation; 0.4–0.6, moderate correlation; 0.2–0.4, weak correlation; and 0.0–0.2, very weakly correlated or unrelated [12].

## Results

### Comparison of PET/CT imaging in patients with different types of NSCLC

**Fig. 1:**
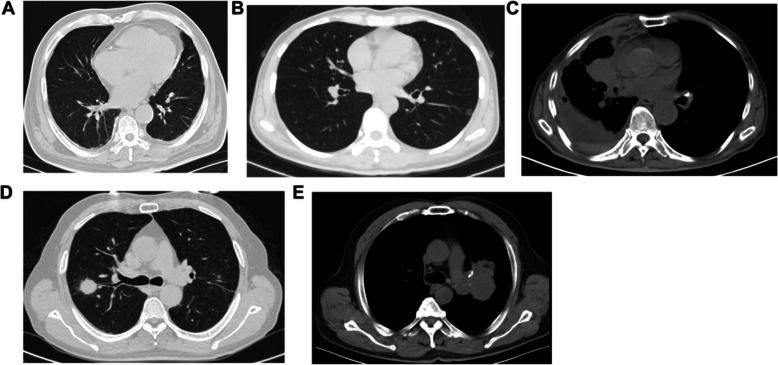
PET/CT imaging in patients with NSCLC. Pulmonary PET/CT in **A** normal individual, **B** patient with early adenocarcinoma, **C** patient with advanced adenocarcinoma, **D** patient with early squamous cell carcinoma, and **E** patient with advanced squamous cell carcinoma

In normal condition of lungs, the bronchovascular bundles were clear; there were no solid infiltrates in the lungs, and no enlarged lymph nodes in the mediastinum and hilum (Fig. 1A). In the lungs of patients with early adenocarcinoma, ground-glass opacity was observed. The boundary was clear, and the vascular bundle sign was visible. Some lesions showed lobulation, burrs, and vacuoles (Fig. 1B). Soft tissue masses were seen in the lungs of patients with advanced adenocarcinoma, associated with tracheal stenosis, mediastinal and hilar lymph node metastasis, and extensive planting metastasis in the ipsilateral pleura (Fig. 1C). In early squamous cell carcinoma, soft tissue masses in the lungs showed lobes and burrs, and the density was uneven (Fig. 1D). In patients with advanced squamous cell carcinoma, the lungs mainly showed local invasion, large soft tissue masses, uneven density, visible lobes and burrs, and visible metastasis of ipsilateral hilar lymph nodes (Fig. 1E).

### Comparison of SUV_max_ in patients with different types of NSCLC

**Fig. 2:**
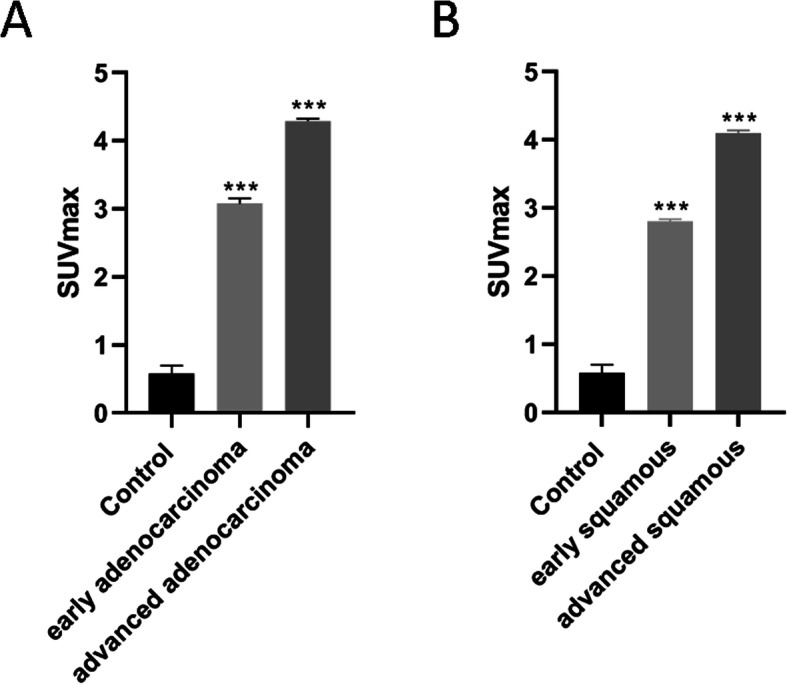
SUV_max_ for patients with different types of NSCLC. **A** Comparison of SUV_max_ between healthy individuals and adenocarcinoma patients. **B** comparison of SUV_max_ between healthy individuals and squamous cell carcinoma patients. ***P < 0.001 vs. control

The SUV_max_ of healthy individuals was ~ 0.6, the SUV_max_ of early adenocarcinoma patients was increased to ~ 3, and the SUV_max_ of patients with advanced adenocarcinoma was increased to ~ 4.3 (Fig. 2A); the difference was significant (*P* < 0.001). The SUV_max_ of healthy individuals was ~ 0.6, the SUV_max_ of early squamous cell carcinoma patients was increased to ~ 2.8, and the SUV_max_ of patients with advanced squamous cell carcinoma was increased to ~ 4.1 (Fig. 2B); the difference was significant (*P* < 0.001). The SUV_max_ in carcinoma tissue was significantly higher than in adjacent area among four groups (*P* < 0.0001, seen in Supplementary Fig.1). The data of tumor site, tumor size, and SUV_max_ for each patient are shown in Supplementary Table 1. SUV_max_ of controls are shown in Supplementary Table 2.

### miRNA-216b expression levels in NSCLC tissues

**Fig. 3:**
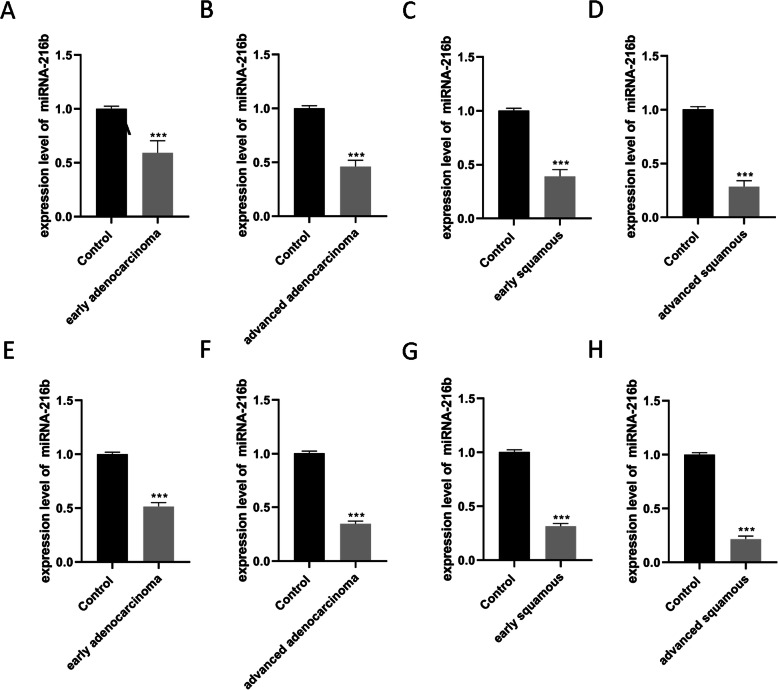
Expression of miRNA-216b in different types of NSCLC. **A E**xpression of miRNA-216b in the plasma of patients with early adenocarcinoma. **B** Expression of miRNA-216b in the plasma of patients with advanced adenocarcinoma. **C** Expression of miRNA-216b in the plasma of patients with early squamous cell carcinoma. **D** The expression of miRNA-216b in the plasma of patients with advanced squamous cell carcinoma. **E** Expression of miRNA-216b in early adenocarcinoma tissue. **F** Expression of miRNA-216b in advanced adenocarcinoma tissue. **G** Expression of miRNA-216b in early squamous cell carcinoma tissue. **H** The expression of miRNA-216b in advanced squamous cell carcinoma. ***P < 0.001 vs. control

The expression of miRNA-216b in plasma was detected. Compared with healthy subjects, the expression of miRNA-216b in the plasma of patients with early adenocarcinoma tissues was reduced to ~ 60% (*P* < 0.001, Fig. 3A), the expression in the plasma of patients with advanced adenocarcinoma tissues was reduced to ~ 46% (*P* < 0.001, Fig. 3B), the expression in the plasma of patients with early squamous cell carcinoma was reduced to ~ 39% (*P* < 0.001, Fig. 3C), and the expression in the plasma of patients with advanced squamous cell carcinoma was reduced to ~ 28% (*P* < 0.001, Fig. 3D).

Then the expression of miRNA-216b in the tissues of patients with small cell lung cancer was detected. Compared with adjacent tissues, the expression of miRNA-216b in early adenocarcinoma tissues was reduced to ~ 51% (*P* < 0.001, Fig. 3E), the expression in advanced adenocarcinoma tissues was reduced to ~ 34% (*P* < 0.001, Fig. 3F), the expression in early squamous cell carcinoma was reduced to ~ 31% (*P* < 0.001, Fig. 3G), and the expression in advanced squamous cell carcinoma was reduced to ~ 21% (*P* < 0.001, Fig. 3H).

### Correlation between SUV_max_ and miRNA-216b expression levels in adenocarcinoma

**Fig. 4:**
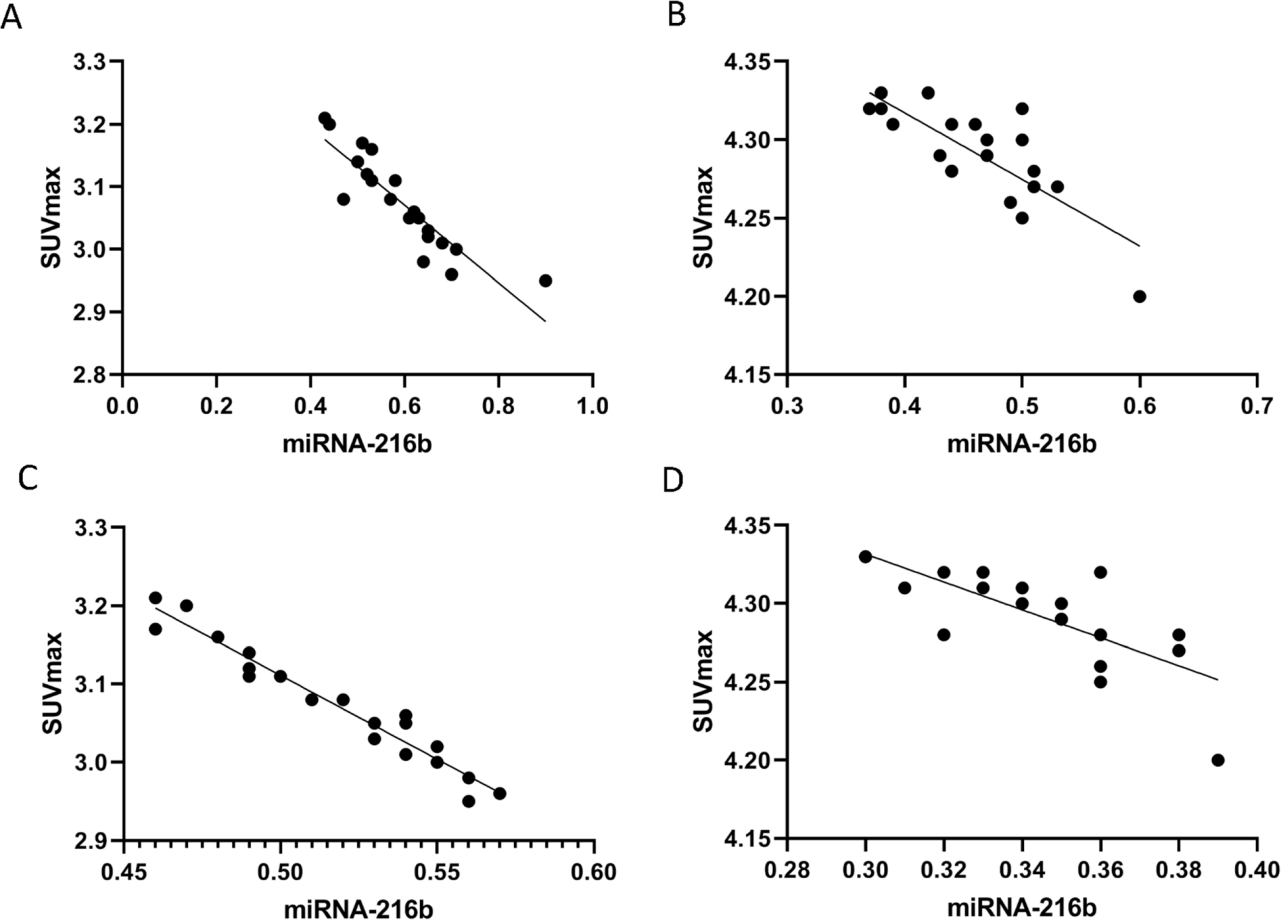
Correlation of SUV_max_ and miRNA-216b expression levels in adenocarcinoma. **A** Correlation analysis between SUVmax and miRNA-216b expression of plasma in early adenocarcinoma. **B** Correlation analysis between SUVmax and miRNA-216b expression of plasma in advanced adenocarcinoma. **C** Correlation analysis between SUVmax and miRNA-216b expression of tissue in early adenocarcinoma. **D** Correlation analysis between SUVmax and miRNA-216b expression of tissue in advanced adenocarcinoma

Pearson correlation analysis showed a negative correlation between SUV_max_ and miRNA-216b expression levels in early and advanced lung adenocarcinoma (Fig. 4A–D).

### Correlation between SUV_max_ and miR-216b expression levels in squamous cell carcinoma

**Fig. 5:**
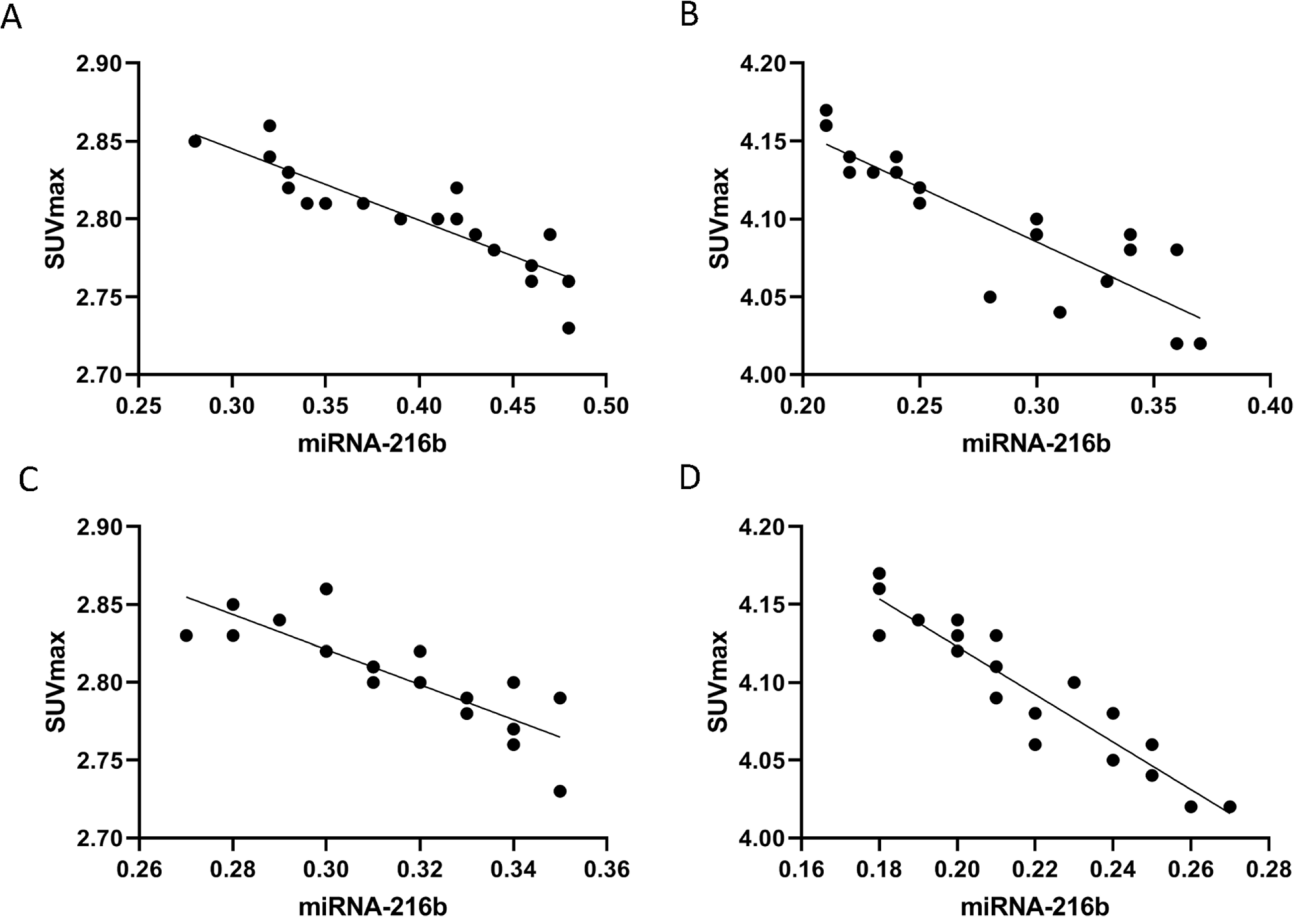
Correlation between SUV_max_ and miRNA-216b expression levels in squamous cell carcinoma. **A** Correlation analysis between SUVmax and miRNA-216b expression of plasma in early squamous cell carcinoma. **B** Correlation analysis between SUVmax and miRNA-216b expression of plasma in advanced squamous cell carcinoma. **C** Correlation analysis between SUVmax and miRNA-216b expression of tissue in early squamous cell carcinoma. **D** Correlation analysis between SUVmax and miRNA-216b expression of tissue in advanced squamous cell carcinoma

Pearson correlation analysis showed a negative correlation between SUV_max_ and miRNA-216b expression levels in early and advanced lung squamous cell carcinoma (Fig. 5A–D).

## Discussion

NSCLC has a poor prognosis and remains the leading cause of cancer death worldwide. Therefore, accurate and effective detection and evaluation are critical [19]. PET/CT is the most commonly used hybrid imaging technology. It has high sensitivity and specificity for detecting metabolic malignant tumors [20–23]. Quantitative assessment of cancer treatment response is an important step in achieving effective and personalized patient care. PET combined with CT using 18F-FDG is a powerful tool for providing predictive information on therapeutic response [24–27].

We found that the SUVmax of patients with adenocarcinoma was higher than that of healthy individuals, and the SUVmax of patients with squamous cell carcinoma was also higher, indicating that the increase in PET/CT SUV value can be used for early detection of non-small cell lung cancer. The expression of SUVmax in patients with advanced adenocarcinoma was higher than that in patients with early adenocarcinoma, and the SUVmax of patients with advanced squamous cell carcinoma was also higher than that of patients with early squamous cell carcinoma, suggesting that the level of PET/CT SUV value can be used for the classification and staging of lung cancer diagnosis. ^18^F-FDG uptake was also a visual representation of energy uptake during malignant proliferation of tumor cells, which helped us better monitor tumor progression and prognosis.

MicroRNAs (miRNAs) are small, single-stranded non-coding RNA molecules that regulate gene expression at the post-transcriptional level. There is growing evidence that miRNAs are aberrantly expressed in many human cancers and play an important role in carcinogenesis and cancer progression. They would interfere with all six major tumor hallmarks: unlimited cell proliferation, autonomous growth, anti-growth inhibition signaling, escape from apoptosis, neoangiogenesis, and tissue invasion and metastatic spread [28, 29]. Peripheral miRNA could be a surrogate of miRNA expression in the tumor biopsy [30]. Recently, circulating and tumor miRNAs are found to be dysregulated in a non-invasive lung cancer [31–33]. And differential expression of specific miRNAs in lung cancer is associated with histological subtyping [34, 35], tumor metastasis [36], and prognostic outcome [37–39]. miRNAs play direct or indirect roles in regulating oncogenes (KRAS), tumor suppressor genes (FHIT, WWOX) [33, 40], and immune-related gene (TLR8) [41], which ultimately promote cancer cell growth and dissemination.

miRNA-216b as a tumor suppressor is downregulated in varieties of cancer types [42]. It was first reported in human nasopharyngeal carcinoma [43]. miRNA-216b-5p was decreased within human breast cancer tissues and was correlated with lymph node metastasis and advanced tumor size, which functioned by targeting HDAC8 [44]. miRNA 216b was also downregulated in pancreatic cancer tissue and appeared to be related with the inhibition of pancreatic cancer cells proliferation as well as a KRAS-silencing induced apoptosis [45]. In addition, miRNA-216b could suppress FoxM1 expression in human glioma, osteosarcoma, liver cancer, cervical cancer, melanoma, and NSCLC [46–51]. Some studies demonstrated that miRNA-216b can inhibit lung cancer cell growth via diverse signal pathways [6, 47, 52, 53] and it was associated with cisplatin sensitivity by modulating autophagy [54–56] and prognosis [57]. These studies implied that miRNA-216b played important role in lung carcinogenesis and dissemination and needed further research, especially in clinical application. In our study, the level of miRNA-216b was detected in plasma and tumor tissue of NSCLC patients. It was found that the levels of miRNA-216b in the plasma and tumor tissues of patients with adenocarcinoma and squamous cell carcinoma were significantly lower than those in healthy people and adjacent tissues, respectively. The levels of miRNA-216b in patients with advanced adenocarcinoma and advanced squamous cell carcinoma were lower than those of patients with early stage adenocarcinoma and early stage squamous cell carcinoma, respectively. Our results suggested that the level of miRNA-216b in patients may be used for the early detection of NSCLC as well as for differentiating between early and advanced cancer. Studies had shown that miRNA-216b can inhibit the proliferation and invasion of NSCLC cells by directly targeting the 3′ untranslated region and negatively regulating the expression of SOX9, which was an oncogene regulated by multiple miRNAs in various types of human cancer [58]. In addition, the upregulation of miRNA-216b expression was related to the histological stage of NSCLC, and patients with lower miRNA-216b levels had a shorter survival time [6]. Liu et al. also demonstrated that serum exosomal miRNA-216b levels were significantly lower in NSCLC patients and were closely associated with poor prognosis [57]. In addition, some studies reported that miRNA-216b was involved in the cisplatin sensitivity by modulating autophagy and apoptosis [52, 54, 56]. Combining these studies, we thought that targeted therapy based on miRNA-216b showed great potential in non-small lung cancer.

Pearson correlation analysis showed that expression levels of SUV_max_ and miRNA-216b were negatively correlated with adenocarcinoma and squamous cell carcinoma patients. We believed that both miRNA-216b expression and ^18^F-FDG uptake could be used as indicators to assess activity of lung lesions and their negative correlation supported the role of detecting miRNA-216b and its possible application in NSCLC. Although our results supported the association between miRNA216b and lung cancer growth, further researches in this field were needed to assess the real value in clinical practice. On the other hand, for clinical transformation, ^18^F-FDG PET/CT could be an indicator to evaluate the therapeutic response and treatment efficacy of targeting miRNA-216b. The miRNA-based tumor therapy, particularly synthetic Mimics, Antigomirs, LNAs, and peptide-conjugated phosphorodiamidate morpholino oligomers (PPMOs), is a new development direction for tumor therapy [59]. However, there are some challenges to apply miRNA in therapeutics. Firstly, the mechanism between miRNA-216b and 18F-FDG uptake is still not clarified, because there are some other unknown factors that could influence their relationship. Next, off-target effects of miRNA-216b might result into more complex biological process, which is the major challenge in the field of miRNA treatment [60]. However, our study contributed to our understanding of the role of miR-216b in the molecular pathogenesis of cancer and therapy as well as the potential value of 18F-FDG PET/CT in the evaluation of miRNA-based therapeutic approaches in NSCLC.

## Conclusion

Our work demonstrated that in patients with NSCLC, miRNA216b expression was reduced, and this phenomenon was correlated with tumor staging. ^18^F-FDG uptake in patients with NSCLC was increased and was correlated with tumor staging; miRNA-216b expression level was negatively correlated with ^18^F-FDG uptake. Hence, our results supported the role of miRNA216b in early detection of lung cancer. In addition, a strong relation between miRNA216 expression and FDG uptake was highlighted. Whether miRNA216b could be adopted as a possible marker to evaluate tumor response in the field of miRNA216b-based cancer treatment as well as its real applicability in clinical practice warrants further investigation.

## Supplementary Information

**Additional file 1: Supplementary Figure 1.** Comparison of SUV_max_ between tumor tissue and adjacent tissues in non-small lung cancer. The SUV_max_ of tumor tissue is significantly higher than adjacent tissue in adenocarcinoma and squamous cell carcinoma.

**Additional file 2: Supplementary Table 1.** Patients and lung cancer characteristics. **Supplementary Table 2.** Values of SUVmax in controls.

## Data Availability

The datasets used and/or analyzed during the current study are available from the corresponding author on reasonable request.
