# Supplementary figures and images for: The expression of miRNA-216b is negatively correlated with 18F-FDG uptake in non-small cell lung cancer
Source: World J Surg Oncol. 2021 Sep 1;19:262. doi: 10.1186/s12957-021-02376-2 (PMC8411519; doi:10.1186/s12957-021-02376-2)

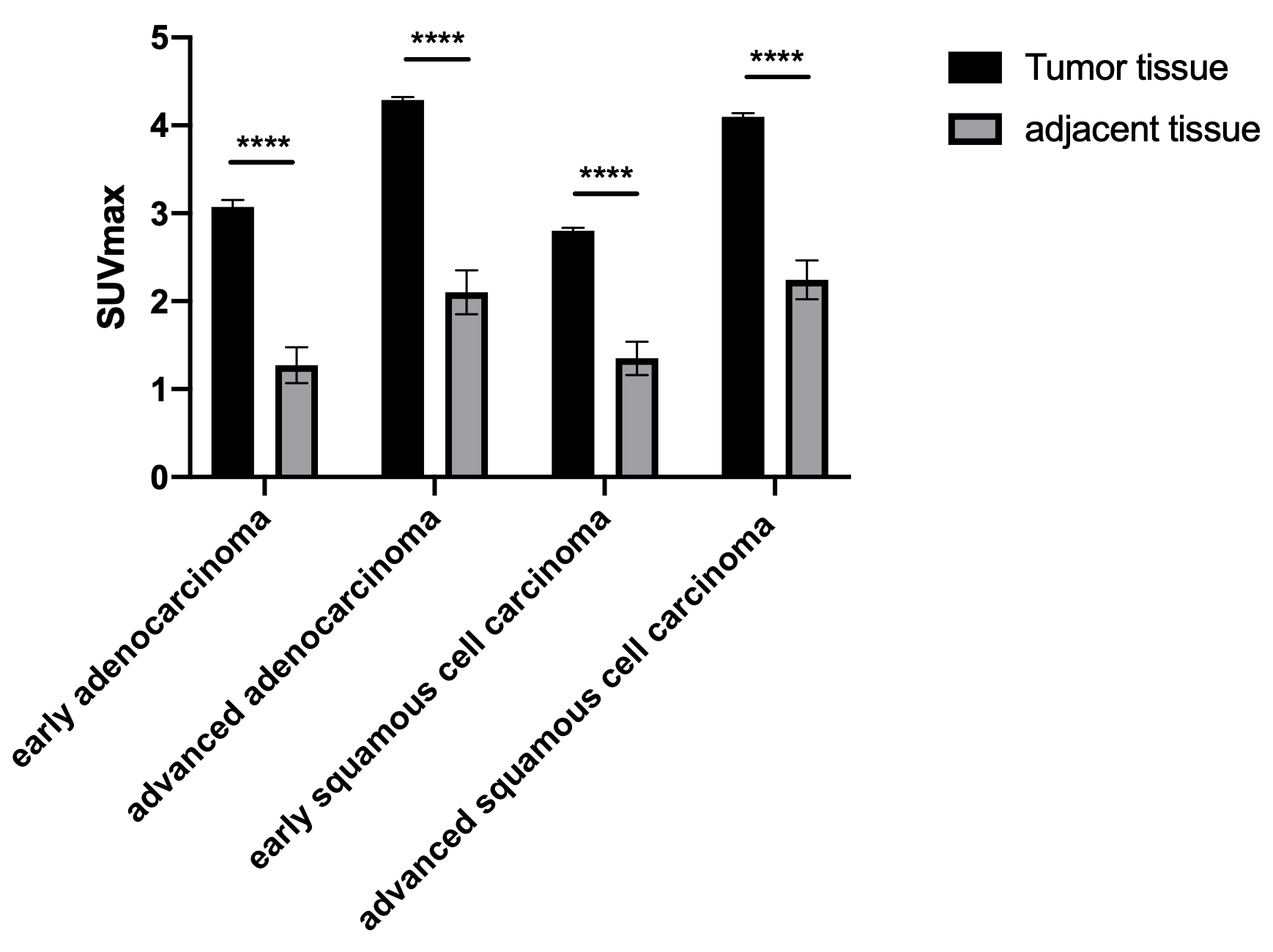

Supplement: Supplementary file 1 — Additional file 1: Supplementary Figure 1. Comparison of SUVmax between tumor tissue and adjacent tissues in non-small lung cancer. The SUVmax of tumor tissue is significantly higher than adjacent tissue in adenocarcinoma and squamous cell carcinoma. [file 12957_2021_2376_MOESM1_ESM.tiff]
