# Supplementary material for: The expression of miRNA-216b is negatively correlated with 18F-FDG uptake in non-small cell lung cancer
Source: World J Surg Oncol. 2021 Sep 1;19:262. doi: 10.1186/s12957-021-02376-2 (PMC8411519; doi:10.1186/s12957-021-02376-2)
Supplement: Supplementary file 2 — Additional file 2: Supplementary Table 1. Patients and lung cancer characteristics. Supplementary Table 2. Values of SUVmax in controls. [file 12957_2021_2376_MOESM2_ESM.docx]

Supplementary Table1 Patients and lung cancer characteristics

| Type | number | location | Tumor size (cm) | SUVmax | Adjacent  SUVmax |
| --- | --- | --- | --- | --- | --- |
| Early ADC | 1 | LU | 1.2 | 3.21 | 1.19 |
| Early ADC | 2 | RU | 3.4 | 3.01 | 1.56 |
| Early ADC | 3 | LL | 2.2 | 2.98 | 1.34 |
| Early ADC | 4 | LL | 2.9 | 2.95 | 1.58 |
| Early ADC | 5 | LU | 4 | 3.2 | 1.03 |
| Early ADC | 6 | RU | 2.9 | 3.11 | 1.3 |
| Early ADC | 7 | RM | 3.2 | 3.17 | 1.29 |
| Early ADC | 8 | RU | 5.2 | 3.03 | 0.95 |
| Early ADC | 9 | LL | 4.3 | 2.96 | 0.99 |
| Early ADC | 10 | RM | 2.8 | 3.16 | 1.3 |
| Early ADC | 11 | RL | 4.8 | 3.14 | 1.26 |
| Early ADC | 12 | LU | 2.4 | 3.05 | 1.72 |
| Early ADC | 13 | RU | 3.7 | 3.08 | 1.31 |
| Early ADC | 14 | RU | 1.8 | 3 | 1.32 |
| Early ADC | 15 | RM | 4.7 | 3.12 | 1.52 |
| Early ADC | 16 | RL | 6.2 | 3.08 | 1.21 |
| Early ADC | 17 | LL | 3.6 | 3.06 | 1.05 |
| Early ADC | 18 | LU | 4.4 | 3.11 | 1.14 |
| Early ADC | 19 | LU | 3.6 | 3.02 | 1.2 |
| Early ADC | 20 | RM | 2.8 | 3.05 | 1.19 |
| Advanced ADC | 21 | RU | 3.2 | 4.32 | 2.05 |
| Advanced ADC | 22 | RU | 2.9 | 4.33 | 1.65 |
| Advanced ADC | 23 | RM | 2.7 | 4.28 | 1.96 |
| Advanced ADC | 24 | RL | 3.7 | 4.3 | 1.82 |
| Advanced ADC | 25 | LL | 4.3 | 4.31 | 2.32 |
| Advanced ADC | 26 | LU | 2.5 | 4.27 | 2.53 |
| Advanced ADC | 27 | RU | 3.6 | 4.28 | 2.61 |
| Advanced ADC | 28 | RU | 1.8 | 4.31 | 2.22 |
| Advanced ADC | 29 | RL | 2.4 | 4.25 | 2.18 |
| Advanced ADC | 30 | LL | 3.3 | 4.33 | 2.13 |
| Advanced ADC | 31 | LL | 4.4 | 4.2 | 2.02 |
| Advanced ADC | 32 | RM | 5.5 | 4.31 | 1.8 |
| Advanced ADC | 33 | RU | 4.8 | 4.29 | 1.9 |
| Advanced ADC | 34 | LU | 1.4 | 4.27 | 2.21 |
| Advanced ADC | 35 | RU | 2.4 | 4.32 | 1.79 |
| Advanced ADC | 36 | RL | 3.6 | 4.3 | 2.1 |
| Advanced ADC | 37 | RM | 2.8 | 4.29 | 2 |
| Advanced ADC | 38 | RM | 6.8 | 4.26 | 2.31 |
| Advanced ADC | 39 | LU | 5.8 | 4.32 | 2.38 |
| Advanced ADC | 40 | RL | 4 | 4.28 | 2.07 |
| Early SCC | 41 | LL | 2.1 | 2.79 | 1.12 |
| Early SCC | 42 | RU | 3.2 | 2.73 | 1.32 |
| Early SCC | 43 | RM | 4.4 | 2.82 | 1.33 |
| Early SCC | 44 | RU | 3.8 | 2.83 | 1.47 |
| Early SCC | 45 | RL | 2.6 | 2.8 | 1.52 |
| Early SCC | 46 | RM | 3.8 | 2.81 | 1.62 |
| Early SCC | 47 | RU | 1.8 | 2.79 | 1.6 |
| Early SCC | 48 | LL | 2.2 | 2.86 | 1.72 |
| Early SCC | 49 | LU | 2.5 | 2.81 | 1.12 |
| Early SCC | 50 | RU | 3.4 | 2.77 | 1.03 |
| Early SCC | 51 | RM | 6.5 | 2.78 | 1.34 |
| Early SCC | 52 | LL | 3.6 | 2.8 | 1.12 |
| Early SCC | 53 | LU | 2.5 | 2.85 | 1.34 |
| Early SCC | 54 | RU | 3.3 | 2.84 | 1.35 |
| Early SCC | 55 | RL | 4.1 | 2.82 | 1.19 |
| Early SCC | 56 | RU | 4.2 | 2.76 | 1.17 |
| Early SCC | 57 | LL | 5.6 | 2.83 | 1.43 |
| Early SCC | 58 | LU | 7.8 | 2.81 | 1.52 |
| Early SCC | 59 | RL | 8.3 | 2.8 | 1.28 |
| Early SCC | 60 | LL | 11.3 | 2.76 | 1.41 |
| Advanced SCC | 61 | LU | 2.2 | 4.17 | 2.5 |
| Advanced SCC | 62 | LL | 1.4 | 4.06 | 2.62 |
| Advanced SCC | 63 | RU | 3.3 | 4.08 | 2.43 |
| Advanced SCC | 64 | RM | 2.9 | 4.13 | 2.41 |
| Advanced SCC | 65 | RU | 4.8 | 4.1 | 2.01 |
| Advanced SCC | 66 | LL | 5.5 | 4.04 | 2.05 |
| Advanced SCC | 67 | RU | 6.7 | 4.02 | 2.43 |
| Advanced SCC | 68 | RM | 8.8 | 4.05 | 2.07 |
| Advanced SCC | 69 | RU | 8.5 | 4.16 | 2.09 |
| Advanced SCC | 70 | LL | 4.2 | 4.14 | 1.97 |
| Advanced SCC | 71 | LU | 1.6 | 4.13 | 2.43 |
| Advanced SCC | 72 | LL | 2.5 | 4.09 | 2 |
| Advanced SCC | 73 | RU | 6.6 | 4.08 | 2.1 |
| Advanced SCC | 74 | RM | 3.7 | 4.11 | 2.24 |
| Advanced SCC | 75 | LL | 4.8 | 4.13 | 2.39 |
| Advanced SCC | 76 | RL | 3.3 | 4.02 | 2.15 |
| Advanced SCC | 77 | RM | 2.6 | 4.09 | 2.25 |
| Advanced SCC | 78 | RL | 3.1 | 4.06 | 1.84 |
| Advanced SCC | 79 | LL | 2.8 | 4.12 | 2.4 |
| Advanced SCC | 80 | LU | 3.8 | 4.14 | 2.51 |

ADC: adenocarcinoma; SCC: squamous cell carcinoma; LU: left upper; LL: left lower; RM: right middle; RU: right upper; RL: right lower

Supplementary Table2 Values of SUVmax in controls

| Number | SUV max |
| --- | --- |
| 1 | 0.62 |
| 2 | 0.6 |
| 3 | 0.58 |
| 4 | 0.1 |
| 5 | 0.55 |
| 6 | 0.64 |
| 7 | 0.6 |
| 8 | 0.61 |
| 9 | 0.63 |
| 10 | 0.62 |
| 11 | 0.59 |
| 12 | 0.65 |
| 13 | 0.58 |
| 14 | 0.56 |
| 15 | 0.62 |
| 16 | 0.63 |
| 17 | 0.61 |
| 18 | 0.6 |
| 19 | 0.61 |
| 20 | 0.62 |
